# Cytoplasmic Location of α1A Voltage-Gated Calcium Channel C-Terminal Fragment (Ca_v_2.1-CTF) Aggregate Is Sufficient to Cause Cell Death

**DOI:** 10.1371/journal.pone.0050121

**Published:** 2013-03-07

**Authors:** Makoto Takahashi, Masato Obayashi, Taro Ishiguro, Nozomu Sato, Yusuke Niimi, Kokoro Ozaki, Kaoru Mogushi, Yasen Mahmut, Hiroshi Tanaka, Fuminori Tsuruta, Ricardo Dolmetsch, Mitsunori Yamada, Hitoshi Takahashi, Takeo Kato, Osamu Mori, Yoshinobu Eishi, Hidehiro Mizusawa, Kinya Ishikawa

**Affiliations:** 1 Department of Neurology and Neurological Science, Graduate School, Tokyo Medical and Dental University, Yushima, Bunkyo-ku, Tokyo, Japan; 2 Information Center for Medical Sciences, Tokyo Medical and Dental University, Yushima, Bunkyo-ku, Tokyo, Japan; 3 Department of Neurobiology, Stanford University School of Medicine, Fairchild Research Building, Palo Alto, California, United States of America; 4 Department of Pathology, Graduate School, Tokyo Medical and Dental University, Yushima, Bunkyo-ku, Tokyo, Japan; 5 Department of Pathology, Pathological Neuroscience Branch, Brain Research Institute, Niigata University, Asahi-machi-dori, Niigata, Japan; 6 Department of Clinical Research, National Hospital Organization, Saigata National Hospital, Saigata, Ohgata-ku, Johetsu-City, Niigata, Japan; 7 Department of Neurology, Hematology, Metabolism, Endocrinology and Diabetology, Yamagata University Faculty of Medicine, Iida-Nishi, Yamagata, Japan; 8 Department of Internal Medicine and Neurology, Hatsuishi Hospital, Nishihara, Kashiwa, Chiba, Japan; University of Waterloo, Canada

## Abstract

The human α_1A_ voltage-dependent calcium channel (Ca_v_2.1) is a pore-forming essential subunit embedded in the plasma membrane. Its cytoplasmic carboxyl(C)-tail contains a small poly-glutamine (Q) tract, whose length is normally 4∼19 Q, but when expanded up to 20∼33Q, the tract causes an autosomal-dominant neurodegenerative disorder, spinocerebellar ataxia type 6 (SCA6). A recent study has shown that a 75-kDa C-terminal fragment (CTF) containing the polyQ tract remains soluble in normal brains, but becomes insoluble mainly in the cytoplasm with additional localization to the nuclei of human SCA6 Purkinje cells. However, the mechanism by which the CTF aggregation leads to neurodegeneration is completely elusive, particularly whether the CTF exerts more toxicity in the nucleus or in the cytoplasm. We tagged recombinant (r)CTF with either nuclear-localization or nuclear-export signal, created doxycyclin-inducible rat pheochromocytoma (PC12) cell lines, and found that the CTF is more toxic in the cytoplasm than in the nucleus, the observations being more obvious with Q28 (disease range) than with Q13 (normal-length). Surprisingly, the CTF aggregates co-localized both with cAMP response element-binding protein (CREB) and phosphorylated-CREB (p-CREB) in the cytoplasm, and Western blot analysis showed that the quantity of CREB and p-CREB were both decreased in the nucleus when the rCTF formed aggregates in the cytoplasm. In human brains, polyQ aggregates also co-localized with CREB in the cytoplasm of SCA6 Purkinje cells, but not in other conditions. Collectively, the cytoplasmic Ca_v_2.1-CTF aggregates are sufficient to cause cell death, and one of the pathogenic mechanisms may be abnormal CREB trafficking in the cytoplasm and reduced CREB and p-CREB levels in the nuclei.

## Introduction

Polyglutamine (polyQ) disease is a group of nine neurodegenerative disorders that are associated with protein aggregation caused by an expansion of the polyQ tract. These disorders include Huntington's disease (HD), spinobulbar muscular atrophy (SBMA), dentatorubral-pallidoluysian atrophy (DRPLA) and spinocerebellar ataxia (SCA) types 1, 2, 3, 6, 7, and 17 (SCA3 is also known as Machado–Joseph disease (MJD)) [1], [2]. In general, the length of the polyQ tract encoded by trinucleotide (CAG) repeat is below 35 in normal individuals. In these diseases, however, the CAG repeat is expanded above 35 to even more than 100, which gives rise to a mutated protein with an expanded polyQ tract that tends to adopt a β-sheet structure, become misfolded, and form oligomers of mutated protein eventually forming microscopic aggregates.

The polyQ expansion causing SCA6 exists in the cytoplasmic carboxyl(C)-tail of the α_1A_ (P/Q-type) voltage-dependent calcium channel protein (Ca_v_2.1) [3]. The cardinal clinical feature of SCA6 is progressive cerebellar ataxia with an average age-of-onset at 45.5 years and gaze-evoked nystagmus [4], [5]. The Purkinje cell of the cerebellar cortex, which expresses Ca_v_2.1 most abundantly in the brain, undergoes degeneration [5], [6]. Previous studies have shown that the polyQ expansion in Ca_v_2.1 causes functional alterations of Ca_v_2.1 [7]–[10]. However, such functional alterations are not considered critical for SCA6 pathogenesis, as Ca_v_2.1 functions were not obviously altered in two independent studies on knock-in mice [11], [12]. Probably more important for the pathogenesis of SCA6 is the formation of microscopic aggregation of Ca_v_2.1, which has been demonstrated in SCA6 human Purkinje cells by using several antibodies against the Ca_v_2.1 C-terminus [6], [13]. SCA6 has several unique features that make it appear as a different disorder among the rest of other polyQ diseases. First, the length of the polyQ tract in the Ca_v_2.1 that is responsible for SCA6 falls within the normal range of repeats for other polyQ diseases (4–19 CAG/polyQs in the Ca_v_2.1 of normal individuals compared with 20–33 CAG/polyQs in SCA6 subjects) [14], [15]. Secondly, microscopic Ca_v_2.1 aggregates can be seen in the cytoplasm (i.e., the cell body or cell processes) of SCA6 Purkinje cells, whereas in other polyQ diseases, aggregates with expanded polyQ are prevalent in the nuclei rather than in the cytoplasm of neurons expressing the responsible proteins [16], [17]. These could indicate that SCA6 has a distinct underlying pathophysiology among polyQ diseases. Recently, a study by Western blot analysis showed that a 75-kDa Ca_v_2.1 C-terminal fragment (CTF), thought to be generated by a proteolytic cleavage of the full-length Ca_v_2.1, might have a critical role in SCA6 pathogenesis from the following reasons [18]. First, the CTF with a normal-length polyQ tract remains soluble and is localized exclusively in the cytosolic fraction of the normal human cerebellum. Second, the CTF becomes insoluble in the cytosolic fraction of SCA6 cerebellum. Third, a small amount of CTF is additionally detected in the nuclear fraction in the human SCA6 cerebellum, suggesting that the expansion of polyQ causes the CTF to translocate into the nucleus as well as to aggregate in the cytoplasm. These findings raise a fundamental question: where (i.e., the nucleus or the cytoplasm) does the CTF exert serious toxicity? In this context, two previous studies reported that a recombinant (r)CTF, when expressed in cultured cells, tends to localize into the nuclei and exert toxicity in the nucleus rather than in the cytoplasm [19], [20]. However, two other studies demonstrated completely opposite data that the rCTF predominantly locates in the cytoplasm where it exerts toxicity [18], [21]. We therefore investigated the relationship between the location of CTF and cell death by using newly created cultured cell models. In addition, we also pursued alterations in protein expression and intracellular localization of the cAMP response element-binding protein (CREB) suggested by the pathogenesis of other polyQ diseases. We finally asked whether the findings in cultured cells are relevant to the human SCA6 pathology. Here we show that the CTF with expanded polyQ is sufficient to cause toxicity in the cytoplasm.

## Results

### Nuclear localization signal (NLS) and nuclear export signal (NES) faithfully targeted the CTF to the desired intracellular locations

**Figure 1:**
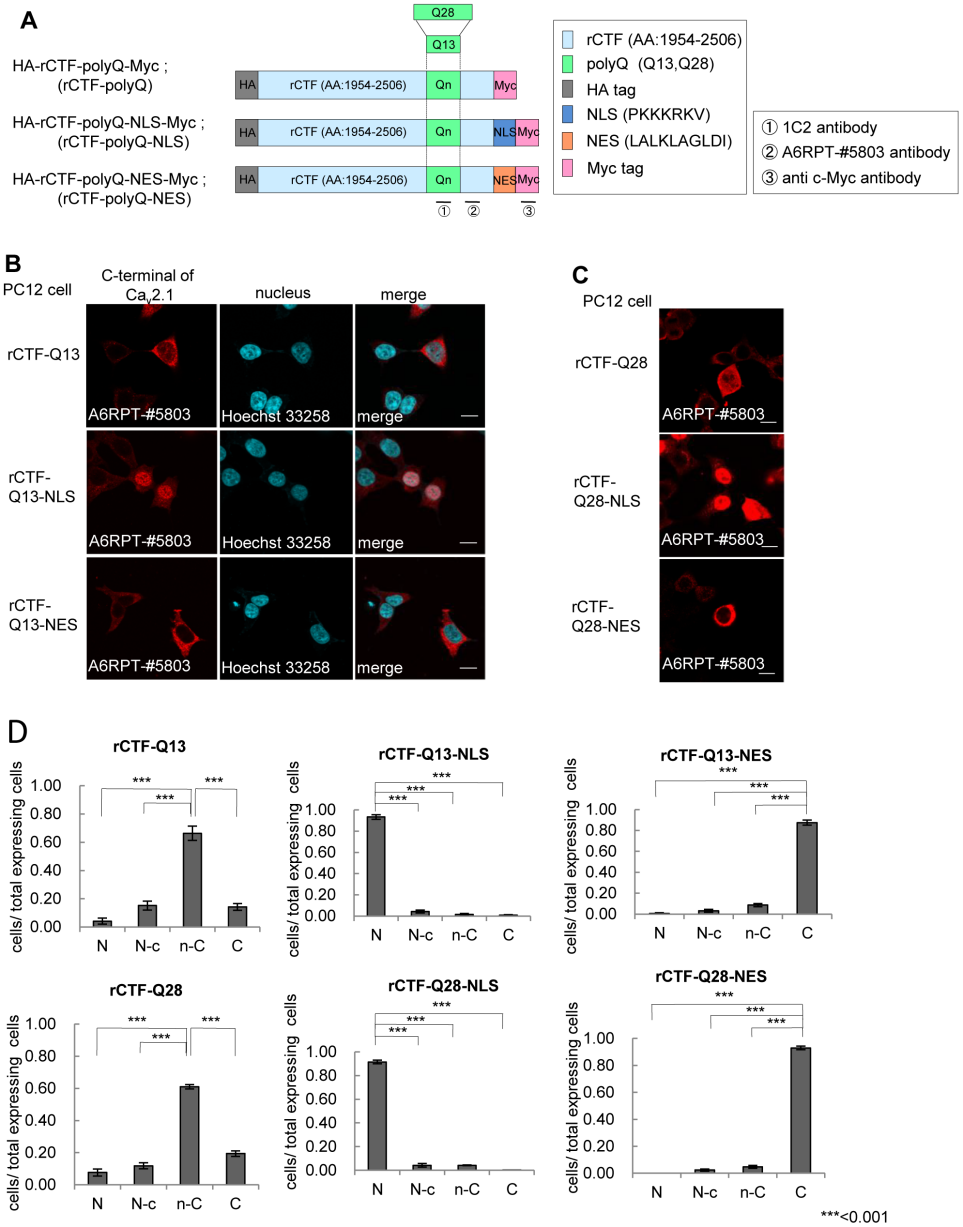
Localization signals efficiently change the intracellular location of CTF. (A) Recombinant Ca_v_2.1 C-terminal fragment (rCTF)s used in this study. Definition of rCTF (amino acid(AA) #1954–2506 in Ca_v_2.1 [GenBank AB035726] [18]), artificial nuclear localization or export signals (NLS or NES), two different polyglutamine (Qn) (Q13; Q28) and recognition sites of three different antibodies (1C2 against expanded polyQ, A6RPT-#5803 against Ca_v_2.1 CTF, c-Myc antibody against Myc-tag) are shown. (B) The rCTF-Q13 is predominantly expressed in the cytoplasm of PC12 cells. The NLS and NES efficiently localize the tagged rCTF to the designed location [A6RPT-#5803 antibody (red), Hoechst 33258 (blue)] (scale bar: 10 µm). (C) The NLS and NES efficiently shift rCTFs with expanded polyQ (Q28) into the nucleus and cytoplasm respectively (scale bars: 10 µm). (D) The proportion of the subcellular localization in each rCTF expressed. The rCTF-polyQ is predominantly, though not exclusively, expressed in the cytoplasm of PC12 cells. The localization signals change subcellular localization of rCTFs-polyQ effectively. (N; the cells expressing rCTF exclusively in the nucleus; N-c: the cells expressing rCTF predominantly in the nucleus than in the cytoplasm; n-C: the cells expressing rCTF predominantly in the cytoplasm than in the nucleus; C: the cells expressing rCTF exclusively in the cytoplasm) (For D: ***:p<0.001;ANOVA. Error bars indicate ± SEM.).

We first asked the primary location of the rCTF. We avoided using the enhanced green-fluorescent protein (EGFP), since a large proportion of the rCTF-polyQ (either Q13 or Q28) dramatically shifted into the nucleus with the presence of EGFP (Figure S1A, S1B, S1C). Instead, we utilized an artificial nuclear localization signal (NLS) and export signal (NES), to regulate the location of the rCTFs (Figure 1A). Two types of polyQ (Q13: normal, Q28: expanded) were used (Figure 1A). We fused the rCTFs with either NLS or NES, transfected each of these in PC12 cells, and then examined the intracellular distributions of these constructs. The rCTF-Q13, the normal version of CTF with an approximate size of 75–80 kDa [18], was predominantly, though not exclusively, expressed in the cytoplasm. In the presence of the NLS tag, the rCTF-Q13-NLS dramatically translocated to the nucleus, whereas the NES tag made the rCTF-Q13-NES anchor in the cytoplasm (Figure 1B). This result was confirmed in human embryonic kidney (HEK) 293T cells (Figure S2), and was consistent with the location of native CTF in human brain [18]. The expanded version of the CTF, rCTF-Q28 was also expressed mainly in the cytoplasm with some obvious amounts in the nucleus (Figure 1C). In contrast, the rCTF-Q28 tagged with NLS showed much stronger tendency to locate in the nuclei, while the rCTF-Q28 tagged with NES appeared to remain entirely in the cytoplasm, showing that both NLS and NES are efficient signals to make the rCTF confine in a desired location. When the intracellular locations of various rCTFs were rated into 4 groups (N: exclusively seen in the nucleus; N-c: predominantly located in the nucleus; n-C: predominantly located in the cytoplasm; C: exclusively seen in the cytoplasm)(*See,* *Materials and Methods* *for details*), the effects of NLS and NES were confirmed (Figure 1D). This allowed us to examine the roles of CTF in the nucleus and in the cytoplasm separately by using the NLS and NES.

### rCTF-NES/-NLS stably expressed in PC12 cell lines with doxycyclin removal

**Figure 2:**
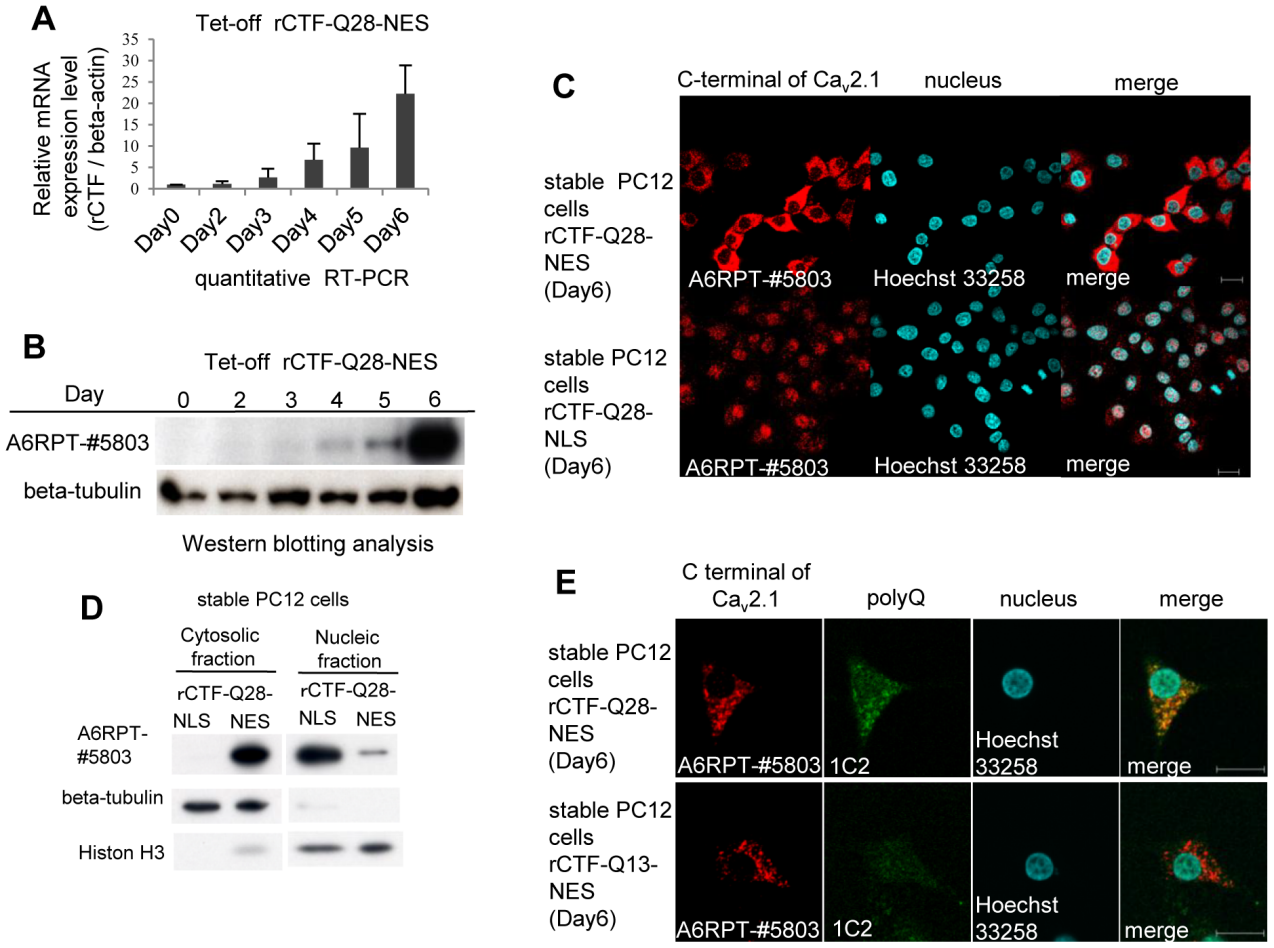
Characterization of inducible PC12 cells stably expressing rCTFs. Only the cell lines expressing rCTF-Q28-NES, rCTF-Q28-NLS and rCTF-Q13-NES are shown here (*Other cells are shown upon request*). (A) A timeline of rCTF expression in PC12 cells stably expressing rCTF-Q28-NES by quantitative real-time PCR (qRT-PCR). The rCTF mRNA level starts to be detected inDay3 and gradually increases with a time-dependent manner. The beta-actin expression level was used as an internal control. (B) A timeline of rCTF expression in PC12 cells stably expressing rCTF-Q28-NES by Western blot using the A6RPT-#5803 antibody. Protein expression starts to be detected on the fourth day after the Dox removal (“Day4”) and reaches abundant level in Day6. Anti beta-tubulin antibody was used as internal control. (C) Immunofluorescence cytochemistry in Day6 stable PC12 cell lines expressing rCTF-Q28-NES (upper row) and rCTF-Q28-NLS (lower row). The NES-tag faithfully anchored rCTF to the cytoplasm, whereas the NLS-tag efficiently directed rCTF to the nucleus. (D) Western blot analysis on protein extracts from stable cell lines expressing rCTF-Q28-NLS and rCTF-Q28-NES confirming efficient intracellular localizations (A6RPT-#5803: anti-Ca_v_2.1CTF, beta-tubulin: a cytoplasmic protein marker, Histone H3: a nuclear protein marker). (E) Immunofluorescence cytochemistry in Day6 stable PC12 cell lines expressing rCTF-Q28-NES (upper row) and rCTF-Q13-NES (lower row). In PC12 cells expressing rCTF-Q28-NES, cytoplasmic aggregates are detected by both A6RPT-#5803 and 1C2. In rCTF-Q13-NES cells, rCTF-aggregates are recognized by the anti-Ca_v_2.1 antibody A6RPT-#5803, but not by 1C2, a monoclonal antibody specific for expanded polyQ. (For C&E, scale bars: 10 µm).

To clarify the chronological sequence of the CTF expression, the formation of CTF aggregates and the point of cell death, we created PC12 cell lines that stably expressed the rCTF by removing the doxycyclin (Dox) (Tet-off PC12 system). In basal conditions when the Dox is kept added in culture medium, Tet-off PC12 cells do *not* express rCTF (termed “Dox(+)” condition). However, when the culture medium was replaced with the one lacking the Dox, cells began to express rCTFs (termed “Dox(−)” condition). Taking an advantage of the fact that the PC12 cells differentiate into cells with neuronal characteristics on exposure to the nerve-growth factor (NGF), we added the NGF and removed the Dox at the same day, which we designate “Day0”. We chose six stable PC12 cell clones (rCTF-Q13, rCTF-Q13-NLS, rCTF-Q13-NES, rCTF-Q28, rCTF-Q28-NLS, rCTF-Q28-NES), which had been confirmed to express equivalent rCTF levels by quantitative real-time reverse-transcription PCR (qRT-PCR) (*data shown upon request*). At the mRNA level, the quantitative RT-PCR showed that the rCTF mRNA level starts to be detected from Day3 (Figure 2A). At the protein level, Western analysis using the A6RPT-#5803 antibody against the C-terminus of Ca_v_2.1 [18] showed that the rCTF expression starts to be detected on the fourth day after Dox removal (“Day4”) and reaches abundant levels by Day6 (Figure 2B). The maximum protein expression level at Day6 was approximately 10% of that expressed in transiently over-expressed PC12 cells (*data shown upon request*).

The fluorescent immunocytochemistry revealed that in the stable PC12 cells expressing rCTF-Q28-NES, the recombinant protein was expressed mainly in the cytoplasm (Figure 2C, upper panels), whereas in stable PC12 cells expressing rCTF-Q28-NLS, the protein was confined to the nucleus (Figure 2C, lower panels). The restricted intracellular distribution by NES and NLS was also confirmed by Western blotting (Figure 2D).

We next searched the timeline of the formation of rCTF aggregates. In PC12 cells expressing rCTF-Q28-NES, the cytoplasmic rCTF aggregates visualized by using A6RPT-#5803 started to be detected on Day4 and become conspicuous on Day6 (Figure 2E). In stable rCTF-Q13-NES cells, the rCTF aggregates were observed only with A6RPT-#5803 but not with 1C2. Because no aggregates are detected by A6RPT-#5803 in normal human brains, the stable PC12 cells expressing rCTF-Q13-NES can be considered as an exaggerated cell model for normal CTF. On the other hand, those expressing rCTF-Q28-NES recapitulated SCA6 aggregates in human Purkinje cells, showing A6RPT-#5803 and 1C2 double-positive aggregates [18].

### Cytoplasmic expression of CTF triggered cell death with aggregate formation

**Figure 3:**
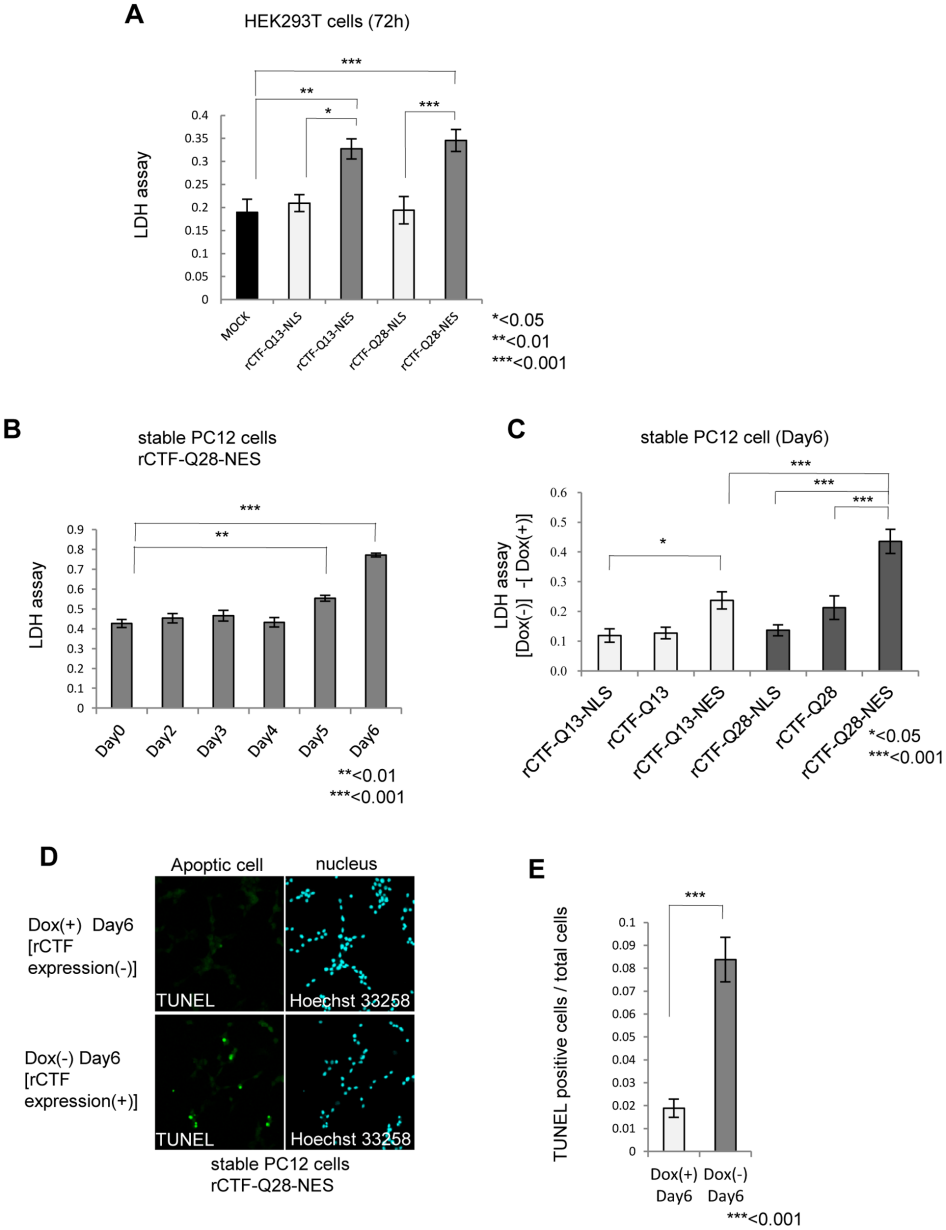
The rCTF is toxic to cells when expressed in the cytoplasm. (A) Cell death in transiently over-expressed HEK293T cells assessed by LDH level at 72 hours after transfection. The rCTF-polyQ-NES exhibits stronger cytotoxicity than rCTF-polyQ-NLS. (B) Time course of cell death in inducible rCTF-Q28-NES PC12 cell line. Cell toxicity is detected from Day5 and become prominent in Day6. (C) The cell death at Day6 compared in 6 inducible PC12 cell lines (rCTF-Q13, rCTF-Q13-NLS, rCTF-Q13-NES, rCTF-Q28, rCTF-Q28-NLS, rCTF-Q28-NES). The cell line expressing rCTF-Q28-NES exerts the strongest cell death. Y-axis shows differences of LDH values between (Dox(−)) and (Dox(+)) states. Cells do not show obvious cell death in Dox(+) states. (D&E) TUNEL positive cells are dramatically increased when doxycyclin is removed (Dox(−)). Eight randomly selected microscope fields were counted for TUNEL positive cells and total cell numbers. (For A to C and E: *: p<0.05; **: p<0.01; ***:p<0.001;ANOVA for A to C. Mann-Whitney's U test for E. Error bars indicate ± SEM.).

We next asked whether the CTF is more toxic in the nucleus than in the cytoplasm, as in other polyQ diseases [2]. To address this question, we transiently over-expressed rCTF-polyQ-NLS or rCTF-polyQ-NES in HEK293T cells, and measured cell death by lactate dehydrogenase (LDH) assay at 72 hours after transfection. Surprisingly, we found that the rCTF-polyQ-NES showed stronger toxicity than the rCTF-polyQ-NLS, regardless of the polyQ lengths (Figure 3A). This is striking, as most proteins causing polyQ diseases show dramatic cell death when they are expressed in the nucleus.

We then compared the cell toxicity for the period after the Dox removal. In the inducible stable PC12 cell line expressing the rCTF-Q28-NES, we found that the cell death measured by LDH assay started to appear on Day5, and become prominent on Day6 (Figure 3B). We also found the cell death is not seen when the Dox is kept in the media, confirming that the cell death is induced by the rCTF expression in the cytoplasm. Based on these results, we compared cell death at Day6 in the 6 stable PC12 cell lines. In a manner consistent with results from transient expression in HEK293T cells, we found that the stable PC12 cells expressing rCTF-polyQ(Q13, Q28)-NES exerted stronger toxicity than rCTF-polyQ(Q13, Q28) or rCTF-polyQ(Q13, Q28)-NLS (Figure 3C). Importantly, we also found that the PC12 cells expressing rCTF-Q28-NES revealed the strongest toxicity among the 6 cell lines. This is an intriguing finding demonstrating that such a small polyQ repeat, the length that corresponds to a normal repeat in other polyQ diseases, exerts toxicity in the cytoplasm of cultured cells. The cell death was compatible with apoptosis and showed a significant increase of TUNEL (TdT-mediated dUTP-biotin nick end labeling)-positive cells (Figure 3D & 3E).

### The cytoplasmic Ca_v_2.1-CTF aggregates co-localize with CREB and phosphorylated(p)-CREB resulting in reductions of CREB/p-CREB in the nuclei

**Figure 4:**
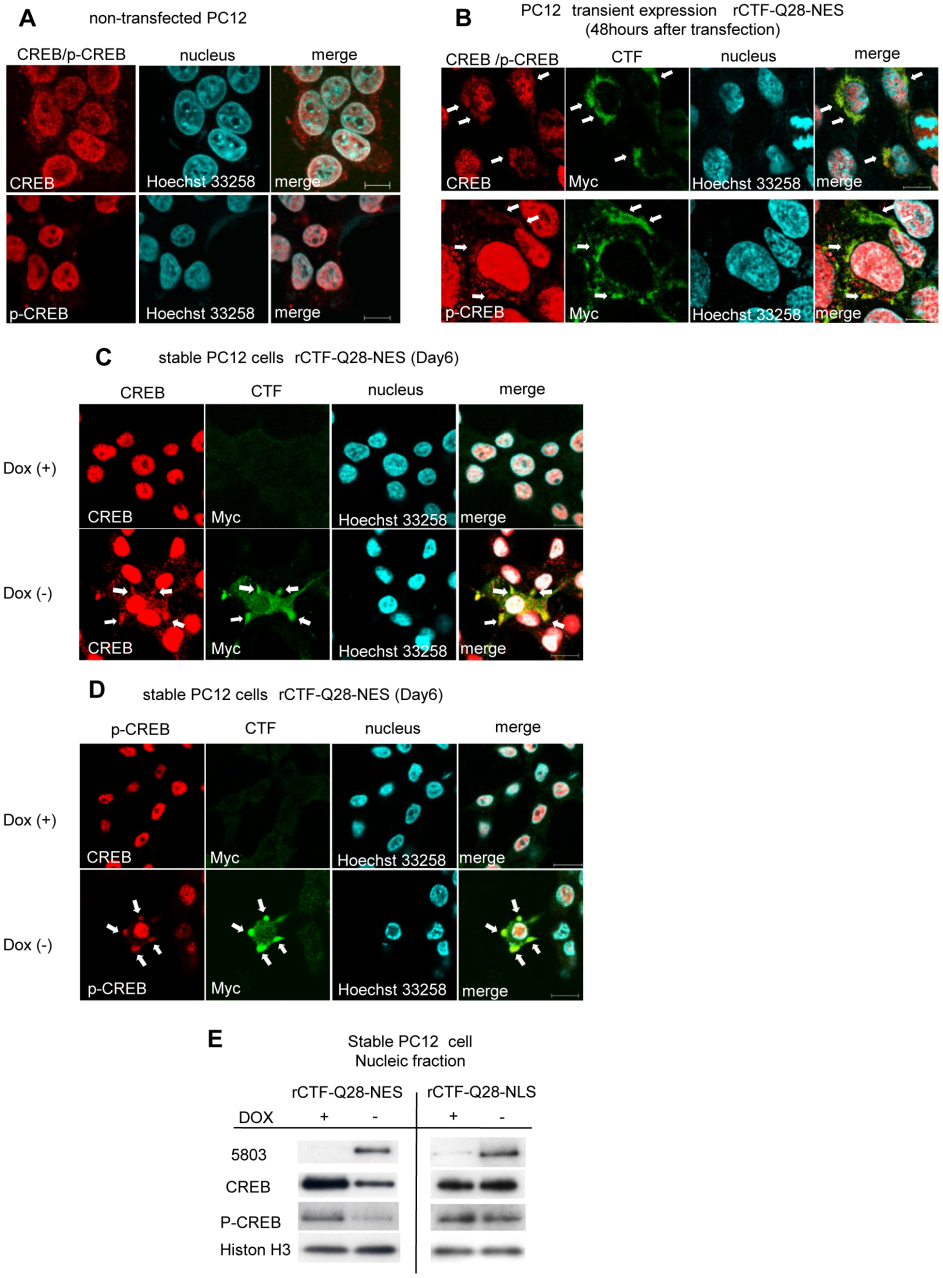
CREB co-localizes with intracytoplasmic CTF aggregates and quantity of CREB is decreased in cultured cell model. (A) In non-transfected PC12 cells, CREB and p-CREB-immunofluorescence labeling is strong in the nucleus, while the cytoplasm shows only weak and diffuse immunofluorescence. (B) In PC12 cells over-expressing rCTF-Q28-NES, the cytoplasmic CTF aggregates co-localize with CREB (*upper row*) and p-CREB (*lower row*), showing focally strong immunofluorescence in their cell bodies (arrows). (C&D) In contrast to the Dox(+) stable PC12 cells (upper row in C&D), co-localizations of CREB (*lower row in C*) or p-CREB (lower row in D) with cytoplasmic aggregates are also observed in Dox(−) stable PC12 cells expressing rCTF-Q28-NES (arrows). (E) The quantities of CREB and p-CREB in the nucleus were both decreased when the rCTF was expressed in the cytoplasm, but not so when the rCTF was targeted in the nucleus. (For A-D: scale bars: 10 µm).

In other polyQ diseases such as HD, one of the underlying mechanisms for suppressing CREB-dependent transcription is the sequestration of CREB-binding protein (CBP) by the aggregations of mutant proteins within the nuclei [22]–[25]. However, it is not known if this is also the case in SCA6. To clarify whether and how the CTF suppresses CREB-dependent transcription, we analyzed localizations of rCTF, CREB and phosphorylated-CREB (p-CREB), the active form of CREB, in PC12 cell models. While the immunoreactivities for CREB and p-CREB were both strong in the nuclei, they were very weak in the cytoplasm of non-transfected PC12 cells (Figure 4A). On the other hand, in PC12 cells transiently expressing rCTF-Q28-NES, we found that the cytoplasmic rCTF aggregates co-localized with both CREB and p-CREB demonstrating focally strong immunoreactivities for these proteins (Figure 4B). Similar co-localizations were also observed both for CREB and p-CREB with rCTF aggregates in the stable PC12 cell lines expressing rCTF-Q28-NES in contrast to the control Dox(+) cells (Figure 4C & 4D). When rCTF-Q13-NES was transiently over-expressed in PC12 cells, CREB and p-CREB also co-localized with rCTF aggregates in the cytoplasm (Figure S3). These results suggested to us that the rCTF expression in the cytoplasm indeed alters intracellular distribution of CREB and p-CREB irrespective of the polyQ length.

We also examined whether the formation of cytoplasmic rCTF aggregates influence the quantity of CREB and p-CREB in the nucleus. By Western blot analysis, we found that they were both decreased when the rCTF was expressed in the cytoplasm (Figure 4E). Surprisingly, the reductions of CREB and p-CREB in the nucleus were not observed when the rCTF was expressed directly in the nucleus. Therefore, over-expressing the rCTF in the cytoplasm leads to the reductions of CREB and p-CREB in the nuclei, possibly by retaining them in the cytoplasm through their co-localization with the CTF aggregates. This also suggests that the cytoplasmic CTF aggregates cause the repression of the CREB-dependent transcription through abnormal intracellular trafficking of CREB.

### CREB co-localized with cytoplasmic aggregates in the human SCA6 Purkinje cells

**Figure 5:**
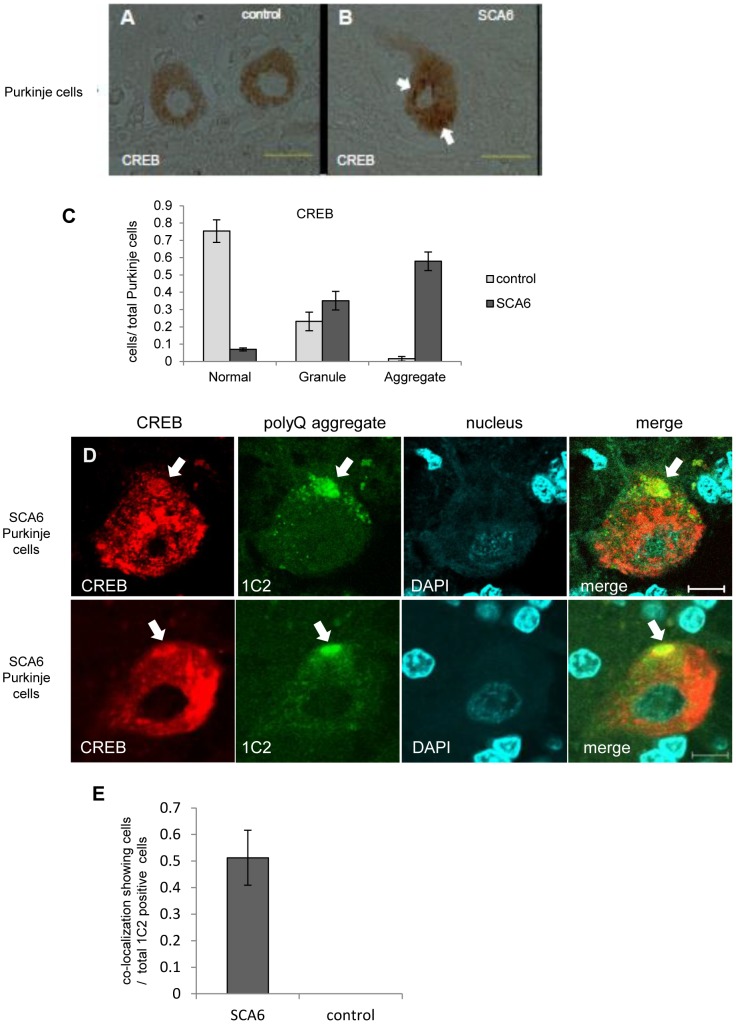
CREB and Ca_v_2.1 aggregates co-localize in SCA6 human Purkinje cells. (A) In a control brain affected with Parkinson's disease, the immunoreactivity against CREB is present but weak and homogeneous in the neuronal cytoplasm of two Purkinje cells. (B) In SCA6 human Purkinje cells, focally accentuated immunoreactive structures (arrows) are seen in the cytoplasm. (C) In ten control brains, a vast majority of Purkinje cells showed weak and diffuse immunoreactivity for the anti-CREB antibody as demonstrated in the Figure 5 A. In contrast, aggregate-like CREB-immunoreactive structures were seen in the Purkinje cells from three SCA6 cerebella. *Please refer to the* *Materials and Methods* *for the detailed description on the criteria of CREB-immunoreactivities.* (D) On double immunofluorescence analysis using a rabbit polyclonal CREB antibody and 1C2, a mouse monoclonal antibody against expanded polyQ tracts, microscopic Ca_v_2.1 aggregates in the cytoplasm of the SCA6 Purkinje cells indeed co-localized with CREB (arrows). (For A&B: scale bars: 50 µm; for D&E: scale bar: 10 µm). (E) In three SCA6 cerebella, approximately 50% of Purkinje cells containing 1C2-positive polyQ aggregates in the cytoplasm showed co-localization of the polyQ aggregates and CREB. In contrast, the Purkinje cells in control cerebella did not show 1C2-positive aggregates resulting in no co-localizations.

We finally examined whether the CREB co-localizes with Ca_v_2.1 microscopic aggregates in SCA6 Purkinje cells. Although CREB expression was seen in the nuclei of cultured cells, the immunoreactivity against CREB was present but weak and homogeneous in the neuronal cytoplasm in control human Purkinje cells (Figure 5A). This would indicate species or cellular differences in the immunoreactivity against the antibody we used. In SCA6 human Purkinje cells, CREB immunoreactivity was also seen expressed in the cytoplasm. However, granular or thread-like CREB-immunoreactive structures were frequently observed in the cytoplasm of SCA6 Purkinje cells (Figure 5B). When these structures were counted, we observed that SCA6 Purkinje cells show a higher number of CREB-immunoreactive structures than controls (Figure 5 C). We next examined the relationship between the CREB and 1C2-immunoreactive Ca_v_2.1 aggregates in the SCA6 Purkinje cells by double immunofluorescence technique. We found that the 1C2-immunoreactive microscopic polyQ aggregates in the cytoplasm of SCA6 Purkinje cells indeed co-localize with CREB (Figure 5D). Among the remaining Purkinje cells in the three SCA6 cerebella, we found that 50% of such Purkinje cells had the co-localization of CREB and Ca_v_2.1 aggregates. As there are no Ca_v_2.1 aggregates in control Purkinje cells, such co-localization is specific to SCA6 Purkinje cells (Figure 5 E). Also, this co-localization mimics what we observed in cultured cells, suggesting that altered intracellular trafficking of CREB takes place in human SCA6 Purkinje cell as well.

## Discussion

Molecular genetics of SCA6 are characterized by two main features; namely, 1) a small polyQ expansion (normal range 4–19 repeat *vs* SCA6 20–33 repeat) that falls within a normal range of other polyQ diseases (usually more than 35 repeat in other polyQ diseases), and 2) the cytoplasmic localization of aggregations which contrasts with nuclear localization in other polyQ diseases [2], [16], [17]. It should be noted that nuclear aggregates were seen in SCA6 Purkinje cells in a very small amount, though cytoplasmic aggregations are far dominant in both immunohistochemical and Western blotting observations [18]. Thus, it remained to be clarified whether cytoplasmic aggregates are more toxic than nuclear ones, or *vice versa* as in other polyQ diseases. To define which location of the CTF is more toxic, and also to elucidate the mechanisms of its toxicity, we developed cell models expressing rCTF tagged with either NLS or NES. These signals faithfully targeted the rCTF to the aimed intracellular cites (cytoplasm or nucleus). We here demonstrate that the rCTF-Q13 and -Q28 both consistently show toxicity in cultured HEK293T and PC12 cells when the rCTFs are expressed in the cytoplasm. On the other hand, neither rCTF-Q13 nor rCTF-Q28 showed obvious toxicity when expressed in the nucleus. Furthermore, the toxicity exerted by rCTF-Q28-NES was more profound than that of rCTF-Q13-NES, an important observation supporting the fundamental idea that SCA6 complies with the general rule of polyQ-length dependent toxicity in all polyQ diseases. Taking these observations together, it seems rational to consider that important cascades in SCA6 pathogenesis occur in the cytoplasm of CTF-expressing cells, such as Purkinje cells.

Then how does the CTF cause cell death? Although this is still an open question, we here showed that the cytoplasmic overexpression of rCTF resulted in the altered intracellular distributions of CREB and p-CREB. The CREB and p-CREB both co-localized with Ca_v_2.1-CTF aggregates in the cytoplasm, and the amount of CREB and p-CREB were both reduced in the nuclei of cultured cells. Furthermore, co-localization of CREB and cytoplasmic Ca_v_2.1 aggregates was confirmed in human SCA6 Purkinje cells, suggesting that the suppression of CREB-mediated transcription takes place in SCA6 Purkinje cells. Indeed, we recently found that the brain derived neurotrophic factor (BDNF) mRNA, which is regulated by the CREB-mediated transcription, is reduced in SCA6 human cerebellum [26]. It is also important to note that the CREB and p-CREB were not reduced even when the rCTF was over-expressed in the nuclei by tagging the rCTF with NLS. Although the present observation is strikingly different than with other polyQ diseases, in which suppression of CREB-transcription is seen by nuclear localizations of mutated proteins [23], [24], [27], it should be noted that polyQ-aggregates are also seen in the cytoplasm in other polyQ diseases such as HD [28] and MJD [13], raising a hypothesis that CREB-transcriptional repression may be further enhanced by the formation of protein aggregates in the cytoplasm. It has been shown that the CREB is transported from axons to cell body [29], and translocated from the cytoplasm to the nucleus [30], [31]. This retrograde trafficking has been claimed to be important for neurons to survive. Thus, it may be possible that the cytoplasmic protein aggregates can affect neuronal survival by trapping the CREB in the cytoplasm resulting in the reductions of the nuclear CREB and pCREB levels. Interestingly, the CREB-related transcription is suppressed in Parkinson's disease by the formation of p-CREB aggregates in the neuronal cytoplasm [31]. However, many issues remain unsolved concerning the link between the Ca_v_2.1 with expanded polyQ and the suppression of the CREB-related transcription. For example, it is not known whether the CTF could directly bind CREB/pCREB, as we failed to detect direct interactions between rCTF and CREB by co-immunoprecipitation experiments (data not shown). It is thus possible that some other proteins mediate co-localization of Ca_v_2.1 aggregates and CREB/pCREB. Further studies are needed to elucidate how CREB and p-CREB co-localize with Ca_v_2.1 aggregates.

Our observation of cytoplasmic toxicity of the CTF is confounded by two previous studies showing rCTF toxicity in the nuclei [19], [20], However, as we showed here, tagging rCTF with EGFP may artificially confine the rCTF into the nuclei. It should also be noted that the components of the rCTFs are not exactly the same as the four studies [18]–[21]: the present study utilized CTF (Amino acids #1954–#2506) [18] which is exactly the same with the one Li et al. used [20], except for the differences in tag proteins. The CTF used by Kordasiewicz HB et al. (Amino acids #2096–#2510) [19]is slightly shorter than ours, and Marquèze-Pouey et al. [21]used a much shorter CTF (corresponding amino acids #2319-C-term of rat Ca_v_2.1). Of note is that the size of rCTF we used here is very close to the native CTF judging from the result in Western blot [18]. The toxicity when expressed in the cytoplasm is also supported by another study showing that the rCTF with expanded polyQ caused cell death and promoted the CTF to be condensed at the plasma membrane by interacting with myosin IIB [21]. Although the rCTF-Q28-NLS was not obviously toxic in our system, we do not preclude a possibility that the CTF in the nucleus could exert subtle toxicity. Since the CTF is also present in the nucleus in the SCA6 human brains, the true pathogenic mechanisms of SCA6 are predicted to be more complicated than our cellular models. Particularly, we must admit that our cultured cells should have different intracellular conditions and thus should respond differently to various stimuli compared to the Purkinje cells. Nevertheless, we consider that tracking downstream events in each location of CTF would be an important step for exploring the disease mechanism.

In conclusion, we showed that the CTF with a small polyQ expansion is sufficient to cause toxicity when it forms aggregates in the cytoplasm. This was associated with changes in CREB/p-CREB intracellular distribution, and their quantities in the nucleus. A precise understanding of the consequences of intracellular CTF aggregations such as the mechanism of CREB inactivation and the downstream gene expression changes appear important for establishing fundamental therapy of SCA6.

## Materials and Methods

### Constructs

Recombinant CTF of Ca_v_2.1 vector (rCTF-polyQ) encoding the C-terminal 553 amino acids (AA) with either 13 or 28 CAG repeats was constructed as previously described [6]. NLS and NES tags were generated from custom oligonucleotides (Sigma-Aldrich Japan, Tokyo, Japan). NLS was derived from SV40 large T antigen (PKKKRKV) and NES was derived from human cAMP-dependent protein kinase inhibitor, alpha form (LALKLAGLDI), and flanked by restriction sites of *Xba*I. The rCTF-polyQ constructs were digested with *EcoR*V and *Pme*I, destroying the *Xba*I restriction site in the multi cloning site. Then, the resulting blunt-end fragment of rCTF-polyQ was ligated into the *Pme*I-digested pcDNA3.1 (Invitrogen, CA, USA). Finally, NLS or NES was cloned into the *Xba*I site of rCTF-polyQ in pcDNA3.1. EGFP fused rCTF vectors were constructed with pEGFP-C2 vectors (Clontech, CA, USA). The rCTF-polyQ constructs were digested with *Bgl*II and *EcoR*V and the resulting fragment of rCTF-polyQ was ligated into EGFP-C2 vector digested with *Bgl*II and *Sma*I in multi cloning site.

### Cell culture and transfection of genes-of-interest

Rat adrenal pheochromocytoma PC12 cells and Human embryonic kidney (HEK293T) cells were obtained from American Type Culture Collection (ATCC, VA, USA). PC12 cells were grown in Dulbecco's Modified Eagle's Medium (DMEM) (WAKO, Saitama, Japan) containing 10% horse serum (HS) (GIBCO, Tokyo, Japan), 5% fetal bovine serum (FBS) (GIBCO) and 1% penicillin/streptomycin (PS) (GIBCO) in a humidified atmosphere of 5% CO_2_ at 37°C. HEK293T cells were grown in DMEM containing 10% FBS and 1% PS in a humidified atmosphere of 5% CO_2_ at 37°C. Cells were transfected using Lipofectamine 2000 (Invitrogen) according to the manufacturer's protocol.

### Generation and culture of inducible PC12 stable cell lines

PC12 Tet-off cell lines (Clontech, CA, USA) were grown in DMEM containing 10% HS, 5% TET-system approved FBS (Clontech), 1% PS and 200 µg/ml G418 (SIGMA, Tokyo, Japan) in a humidified atmosphere of 5% CO_2_ at 37°C. Tet-off PC12 cells were transfected with each rCTF vector at 70% confluence using Lipofectamine 2000 (Invitrogen). Positive clones were selected in complete medium containing 200 µg/ml G418 (SIGMA), 200 µg/ml hygromycin (WAKO) and 2 µg/ml doxycycline (Dox) (Clontech). To initiate differentiation, cells were plated on collagen type I-coated plate (IWAKI, Tokyo, Japan) and grown in DMEM containing 1% HS, 1% PS, 200 µg/ml G418, 200 µg/ml hygromycin, 2 µg/ml Dox and 50 ng/ml nerve growth gactor (NGF) 2.5S (Invitrogen, CA, USA).

### Immunoblot analysis

The procedure was described previously [18]. Subcellular fractionation was performed with NE-PER Nuclear and Cytoplasmic Extraction Reagents (Pierce, IL, USA).

### Cell toxicity and viability analysis

Cell toxicity were assessed by lactate dehydrogenase (LDH) assay with CytoTox 96® Non-Radioactive Cytotoxicity Assay (Promega, WI, USA) and TUNEL assay with DeadEnd^tm^ Fluorometric TUNEL System (Promega). Cell toxicity with LDH assay was calculated using the following formula (experimental LDH release/maximum LDH release).

### Immunocytochemistry

The procedure was described previously [6], [18]. Either of the following primary antibodies was used; mouse monoclonal anti-c-Myc antibody (diluted with phosphate buffered saline (PBS) into 1∶100) (Santa Cruz Biotechnology, CA, USA), rabbit polyclonal A6RPT-#5803 (1∶500), rabbit polyclonal anti-CREB antibody (1∶500) (Cell Signaling Technology, MA, USA), rabbit polyclonal anti-p-CREB antibody (1∶100) (Cell Signaling Technology), mouse monoclonal anti-expanded 1C2 (1∶1000)(Millipore, CA, USA). To visualize the anti-p-CREB antibody signal, endogenous PKA was activated by the 10 µM forskolin containing medium for 1 hour before the fixation.

To examine the proportion of cells with a particular subcellular localization of each rCTF, the cells expressing each rCTF were classified into the following four groups and counted in five randomly selected microscope fields: N; the cells expressing rCTF exclusively in the nucleus; N-c: the cells expressing rCTF predominantly in the nucleus than in the cytoplasm; n-C: the cells expressing rCTF predominantly in the cytoplasm than in the nucleus; C: the cells expressing rCTF exclusively in the cytoplasm. The proportion of subcellular localization was calculated using the following formula: total number of cells in each of the four groups/total rCTF expressing cells. The difference was then statistically analyzed using ANOVA.

### Immunohistochemistry

Formalin-fixed paraffin-embedded tissue sections of the cerebellar cortex were analyzed. Three SCA6 brains and 10 controls (4 individuals with Parkinson's disease, 2 with amyotrophic lateral sclerosis, 2 with multiple system atrophy, 1 with cerebral infarction, and 1 with SCA31) were investigated. The procedure was described previously [6], [13], [18]. Either of the following primary antibodies was used; A6RPT-#5803 (diluted in 1∶500 concentration with PBS), 1C2 (1∶1000), and anti-CREB antibody (1∶100). For double immunofluorescent labeling, sections were incubated with 1% sudan black B in 70% methanol for 5 minutes to block the autofluorescence.

To examine the proportion of Purkinje cells which shows different immunostaining against CREB, five random microscopic fields were selected and each immunostained Purkinje cell was classified into the following three categories; “Normal”: the Purkinje cells which were stained diffusely in the cytoplasm; “Granule”: the Purkinje cells containing small puncta in the cytoplasm; “Aggregate”: the Purkinje cells showing thread-like structures or coarse large aggregates in the cytoplasm. The proportion of each category was calculated using the following formula: the number of Purkinje cell in each of the three groups/total number of immunostained Purkinje cells.

To examine the proportion of Purkinje cells which shows co-localization of CREB and 1C2 positive aggregates, ten consecutive Purkinje cells containing 1C2 positive aggregates in the cytoplasm was examined under a confocal laser microscope in three different regions of cerebellar cortex and calculated.

### RNA isolation and real time quantitative PCR (RT-qPCR)

Total cellular RNA was isolated by TRIzol (Invitrogen, CA, USA) according to the manufactures' protocol. Cellular extracts were then treated with DNase (Invitrogen) and total RNA was quantified on a Nanodrop spectrophotometer. Total RNA was carried out reverse transcription to cDNA using a super script® III first-strand synthesis system for RT-PCR (Invitrogen) and random hexamers. The primer and probes for rCTF was designed from Life technologies (Applied biosystems by Life technologies, CA, USA). The other primer and probes were from TaqMan® Gene Expression Assays.

### Statistical analysis

Where applicable, data are presented as mean ±standard error of the mean (SEM), and statistical analysis was tested by using Mann-Whitney's U test (for TUNEL assay) or a one-way analysis of variance (ANOVA) tests (all assays except for TUNEL assay). Each experiment was repeated three times independently and statistical analysis was performed.

## Supporting Information

Figure S1 Presence of enhanced green fluorescent protein (EGFP) dramatically shifts the tagged rCTF into the nuclei. (A) A scheme of the recombinant Ca_v_2.1 C-terminal fragment (rCTF) vectors with or without EGFP used in this study. (B) The rCTF-polyQ (either Q13 or Q28)-EGFP locates almost exclusively in the nuclei irrespective of the length of polyQ. This localization is quite different from the predominantly cytoplasmic location of rCTF-polyQ without EGFP. (scale bars: 50μm) (C) The proportion of the subcellular localization of each rCTF in the transiently expressing PC12 cells. (N; the cells expressing rCTF exclusively in the nucleus; N-c: the cells expressing rCTF predominantly in the nucleus than in the cytoplasm; n-C: the cells expressing rCTF predominantly in the cytoplasm than in the nucleus; C: the cells expressing rCTF exclusively in the cytoplasm).
(PPTX)

Figure S2 rCTF expression in HEK293T cells. While rCTF-Q13 distributed both in the cytoplasm and nucleus of HEK293T cells, NLS and NES both efficiently shifted the rCTF localization in the nucleus and cytoplasm, respectively. (scale bars: 10μm).
(PPTX)

Figure S3 Co-localization of CREB and p-CREB with cytoplasmic aggregates in rCTFQ13-NES expressing PC12 cells. In PC12 cells over-expressing rCTF-Q13-NES, some of the cytoplasmic CTF aggregates co-localized with CREB (upper row) and p-CREB (lower row) (co-localizations: arrows).
(PPTX)
