# Supplementary material for: Cytoplasmic Location of α1A Voltage-Gated Calcium Channel C-Terminal Fragment (Cav2.1-CTF) Aggregate Is Sufficient to Cause Cell Death
Source: PLoS One. 2013 Mar 7;8(3):e50121. doi: 10.1371/journal.pone.0050121 (PMC3591409; doi:10.1371/journal.pone.0050121)
Supplement: Figure S1 — Presence of enhanced green fluorescent protein (EGFP) dramatically shifts the tagged rCTF into the nuclei. (A) A scheme of the recombinant Cav2.1 C-terminal fragment (rCTF) vectors with or without EGFP used in this study. (B) The rCTF-polyQ (either Q13 or Q28)-EGFP locates almost exclusively in the nuclei irrespective of the length of polyQ. This localization is quite different from the predominantly cytoplasmic location of rCTF-polyQ without EGFP. (scale bars: 50μm) (C) The proportion of the subcellular localization of each rCTF in the transiently expressing PC12 cells. (N; the cells expressing rCTF exclusively in the nucleus; N-c: the cells expressing rCTF predominantly in the nucleus than in the cytoplasm; n-C: the cells expressing rCTF predominantly in the cytoplasm than in the nucleus; C: the cells expressing rCTF exclusively in the cytoplasm). (PPTX) [file pone.0050121.s001.pptx]

## Slide 1
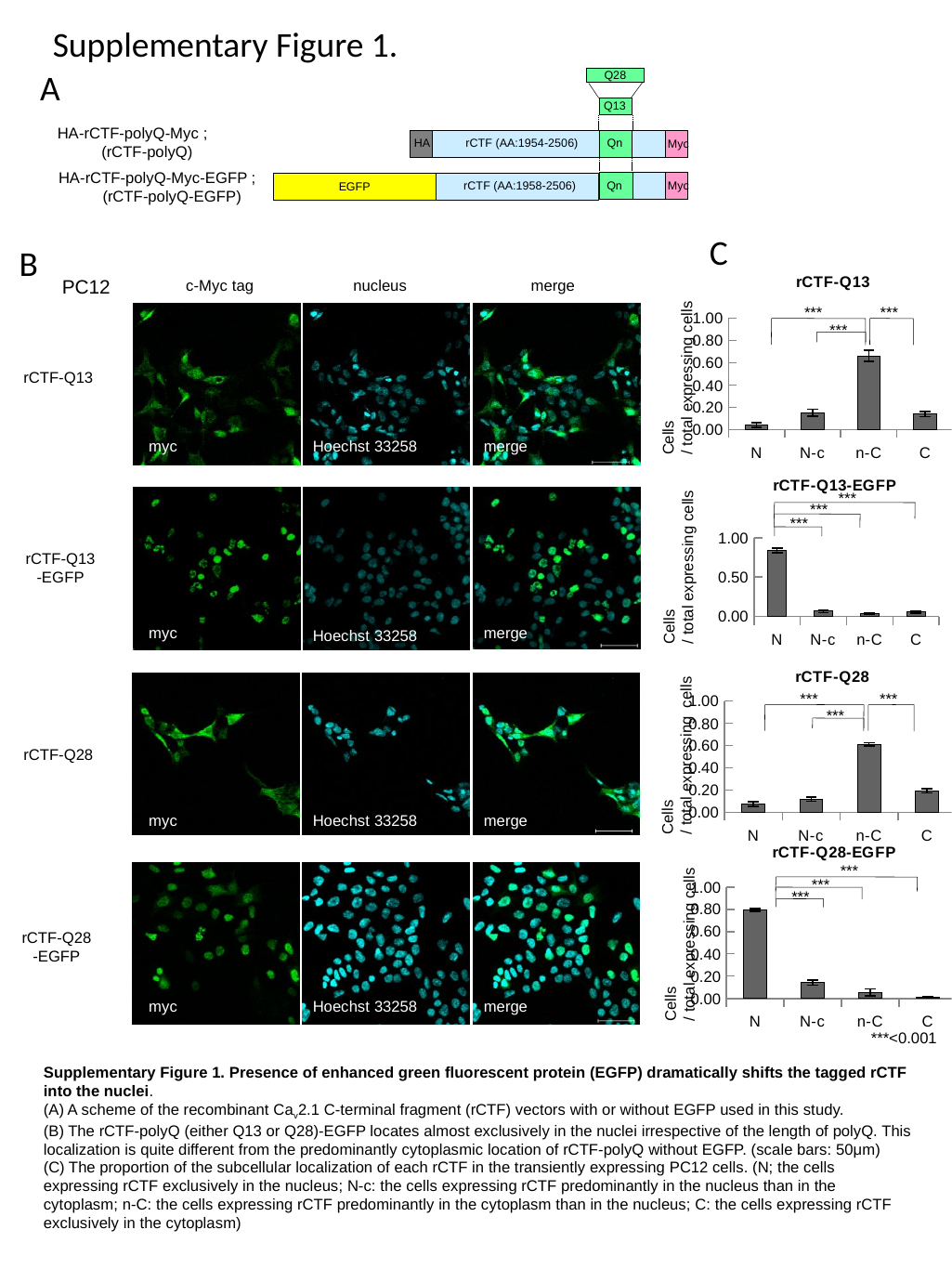

Supplementary Figure 1.
A
Q28
Q13
Qn
HA
rCTF (AA:1954-2506)
Myc
Qn
rCTF (AA:1958-2506)
Myc
EGFP
HA-rCTF-polyQ-Myc ;
 (rCTF-polyQ)
HA-rCTF-polyQ-Myc-EGFP ;
 (rCTF-polyQ-EGFP)
C
B
PC12
### Chart: rCTF-Q13
| Category | |
|---|---|
| N | 0.041791976040432034 |
| N-c | 0.15238347008173744 |
| n-C | 0.663483816372378 |
| C | 0.14234073750546103 |Cells
/ total expressing cells
c-Myc tag
nucleus
merge
***
***
***
rCTF-Q13
myc
Hoechst 33258
merge
### Chart: rCTF-Q13-EGFP
| Category | |
|---|---|
| N | 0.8406396360995121 |
| N-c | 0.06630600507269674 |
| n-C | 0.03718767787184922 |
| C | 0.0558666809559541 |Cells
/ total expressing cells
***
***
***
rCTF-Q13
-EGFP
myc
merge
Hoechst 33258
### Chart: rCTF-Q28
| Category | |
|---|---|
| N | 0.07685478084371451 |
| N-c | 0.11841972685534055 |
| n-C | 0.6105972496968477 |
| C | 0.19412824260409772 |Cells
/ total expressing cells
rCTF-Q28
myc
Hoechst 33258
merge
***
***
***
### Chart: rCTF-Q28-EGFP
| Category | |
|---|---|
| N | 0.7929096044183989 |
| N-c | 0.14302329595569171 |
| n-C | 0.05470231787179753 |
| C | 0.009364781754119027 |Cells
/ total expressing cells
***
***
***
rCTF-Q28
-EGFP
myc
Hoechst 33258
merge
***<0.001
Supplementary Figure 1. Presence of enhanced green fluorescent protein (EGFP) dramatically shifts the tagged rCTF into the nuclei.
(A) A scheme of the recombinant Cav2.1 C-terminal fragment (rCTF) vectors with or without EGFP used in this study.
(B) The rCTF-polyQ (either Q13 or Q28)-EGFP locates almost exclusively in the nuclei irrespective of the length of polyQ. This localization is quite different from the predominantly cytoplasmic location of rCTF-polyQ without EGFP. (scale bars: 50μm)
(C) The proportion of the subcellular localization of each rCTF in the transiently expressing PC12 cells. (N; the cells expressing rCTF exclusively in the nucleus; N-c: the cells expressing rCTF predominantly in the nucleus than in the cytoplasm; n-C: the cells expressing rCTF predominantly in the cytoplasm than in the nucleus; C: the cells expressing rCTF exclusively in the cytoplasm)
