# Supplementary material for: Cytoplasmic Location of α1A Voltage-Gated Calcium Channel C-Terminal Fragment (Cav2.1-CTF) Aggregate Is Sufficient to Cause Cell Death
Source: PLoS One. 2013 Mar 7;8(3):e50121. doi: 10.1371/journal.pone.0050121 (PMC3591409; doi:10.1371/journal.pone.0050121)
Supplement: Figure S2 — rCTF expression in HEK293T cells. While rCTF-Q13 distributed both in the cytoplasm and nucleus of HEK293T cells, NLS and NES both efficiently shifted the rCTF localization in the nucleus and cytoplasm, respectively. (scale bars: 10μm). (PPTX) [file pone.0050121.s002.pptx]

## Slide 1
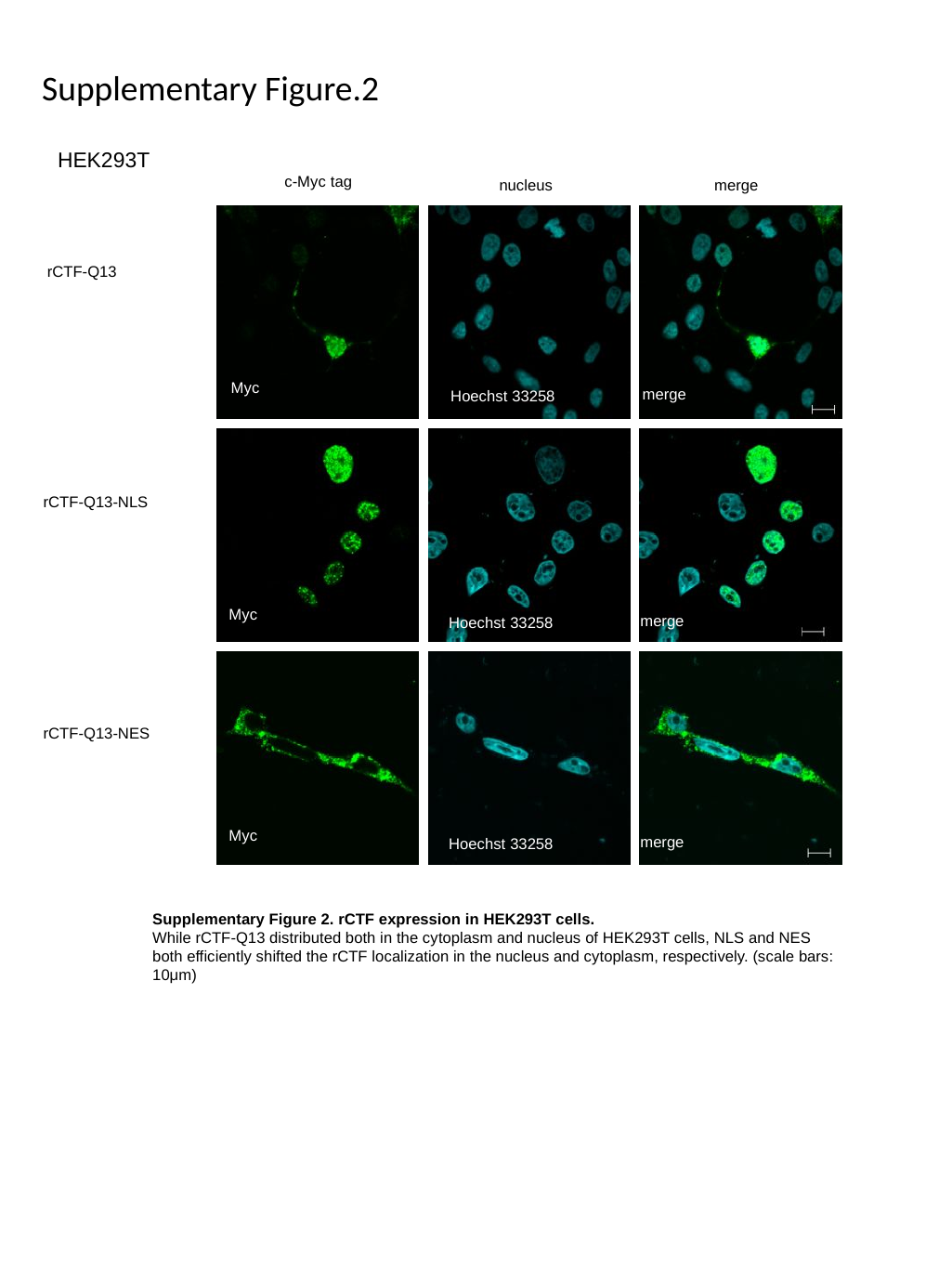

Supplementary Figure.2
HEK293T
c-Myc tag
nucleus
merge
rCTF-Q13
Myc
merge
Hoechst 33258
rCTF-Q13-NLS
Myc
merge
Hoechst 33258
rCTF-Q13-NES
Myc
merge
Hoechst 33258
Supplementary Figure 2. rCTF expression in HEK293T cells.
While rCTF-Q13 distributed both in the cytoplasm and nucleus of HEK293T cells, NLS and NES both efficiently shifted the rCTF localization in the nucleus and cytoplasm, respectively. (scale bars: 10μm)
