# Supplementary material for: Cytoplasmic Location of α1A Voltage-Gated Calcium Channel C-Terminal Fragment (Cav2.1-CTF) Aggregate Is Sufficient to Cause Cell Death
Source: PLoS One. 2013 Mar 7;8(3):e50121. doi: 10.1371/journal.pone.0050121 (PMC3591409; doi:10.1371/journal.pone.0050121)
Supplement: Figure S3 — Co-localization of CREB and p-CREB with cytoplasmic aggregates in rCTFQ13-NES expressing PC12 cells. In PC12 cells over-expressing rCTF-Q13-NES, some of the cytoplasmic CTF aggregates co-localized with CREB (upper row) and p-CREB (lower row) (co-localizations: arrows). (PPTX) [file pone.0050121.s003.pptx]

## Slide 1
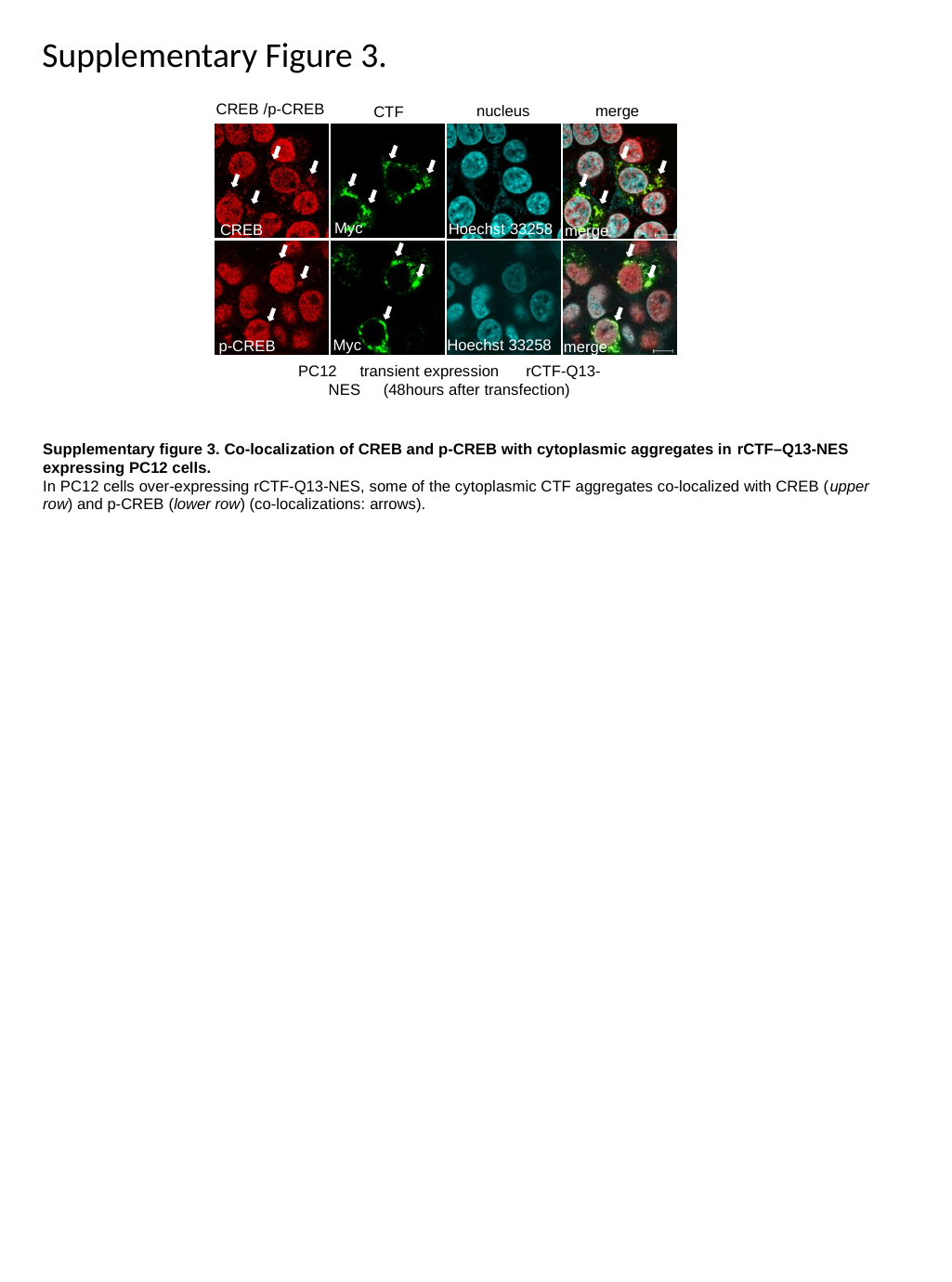

Supplementary Figure 3.
CREB /p-CREB
nucleus
merge
CTF
Myc
Hoechst 33258
CREB
merge
Myc
Hoechst 33258
p-CREB
merge
PC12　transient expression　 rCTF-Q13-NES　(48hours after transfection)
Supplementary figure 3. Co-localization of CREB and p-CREB with cytoplasmic aggregates in rCTF–Q13-NES expressing PC12 cells.
In PC12 cells over-expressing rCTF-Q13-NES, some of the cytoplasmic CTF aggregates co-localized with CREB (upper row) and p-CREB (lower row) (co-localizations: arrows).
